# International citizen project to assess early stage adherence to public health measures for COVID-19 in South Africa

**DOI:** 10.1371/journal.pone.0248055

**Published:** 2021-03-04

**Authors:** Mohammed Majam, Alex Fischer, Jane Phiri, Francois Venter, Samanta T. Lalla-Edward

**Affiliations:** Ezintsha, Faculty of Health Sciences, University of the Witwatersrand, Johannesburg, South Africa; University of Toronto, CANADA

## Abstract

**Introduction:**

With over 500 000 infections and nearly 12 000 deaths, South Africa (SA) is the African epicenter of the current Coronavirus (COVID-19) pandemic. SA has implemented a 5-stage Risk-Adjusted Strategy which includes a phased national lockdown, requiring social distancing, frequent hand washing and wearing face masks. Strict adherence to this strategy is crucial to reducing COVID-19 transmission, flattening the curve, and preventing resurgence. As part of the 22-country International Citizens Project COVID-19 (ICPcovid), this study aimed to describe the SA adherence to the Risk-Adjusted Strategy and identify determinants of adherence.

**Method:**

During 24 April-15 May 2020, people were electronically invited, through social media platforms and a text blast, to complete an online survey, accessible via www.icpcovid.com. The survey investigated COVID-19 testing and preventative adherence measures, then used logistic regression analysis to identify predictors of adherence.

**Results:**

There were 951 participants, with 731(76.9%) 25 to 54 years. Most (672;70.7%) were female, and 705(74.1%) had a university degree. Since the epidemic started, 529(55.6%) and 436(45.9%) participants stated they were eating healthier and taking more vitamins, respectively. Only 82(8.6%) had been COVID-19 tested, and 1(1.2%) tested positive. In public, 905(95.2%) socially distanced, however 99(10.4%) participants had recently attended meetings with over ten people. Regular hand washing was practiced by 907(95.4%) participants, 774(81.4%) wore face masks and 854(89.8%) stayed home when they experienced flu-like symptoms. The odds of adhering to the guidelines were lower among men versus women (AOR 0.72, 95% confidence interval [CI] = 0.528, 0.971) and those who had flu-like symptoms (AOR 0.42, 95% CI = 0.277, 0.628). In contrast, increased odds were reported for those who reported increased vitamin intake (AOR 1.37, 95% CI = 1.044,1.798), and were either cohabiting or married (AOR 1.39, 95% CI = 1.042,1.847).

**Conclusion:**

Despite high reported adherence, face mask use and symptomatic individuals not self-isolating, were areas for improvement. However, these factors cannot solely account for SA’s increasing COVID-19 cases. Larger general population studies are needed to identify other adherence predictors for a strengthened SA COVID-19 response. While the government must continue to educate the entire population on preventative measures, provide personal protective equipment and stress the importance of adherence, there also needs to be implementation of prioritised prevention strategies for men and single individuals to address their demonstrated lower adherence.

## Introduction

The Severe Acute Respiratory Syndrome Coronavirus 2 (commonly referred to as COVID-19) is a respiratory disease that was first documented as an unknown pneumonia outbreak in Hubei Province, China, in December 2019 [1, 2]. COVID-19 is highly contagious, and the disease has overwhelmingly spread worldwide; as of 16 August 2020, there have been more than 2 million deaths and over 105 million infections, across 216 countries and territories. While the United States is currently the global epicentre with over 26 million infections, South Africa is the African epicentre, with over 1.5a million infections and 46 180 deaths [3]. The majority of infected adults only experience mild flu-like symptoms; however, people experience severe illness, and hospitalization where mechanical ventilation may be required, especially those with variant strains, co-morbidities and/or above the age of 65 [4, 5].

The transmission of COVID-19 is primarily through respiratory droplets via direct, indirect, or close contact with an infected person, even when the infected person is asymptomatic or when the droplets remain on contaminated objects [6, 7]. This type of transmission quickly spreads through populations, and to slow the spread, the WHO has recommended interventions to reduce the contact between infected and uninfected people [8]. These interventions include frequent hand washing, face mask use, national lockdowns, social distancing and self-isolation, and many countries have adopted these measures [9]. In late March 2020, South Africa introduced its 5-stage COVID-19 Risk Adjusted Strategy, which began as a 21-day level 5 lockdown, and as of July 30, 2020 the country was still in a level 3 lockdown, while reporting more than 11 000 new cases a day [10, 11].

During the level-5 lockdown, only essential services, such as healthcare facilities, pharmacies and grocery stores remained open, and all citizens were instructed to remain home, to curb community transmission of the disease. Essential personnel, such as healthcare workers and police, could leave their homes for work, but the rest of the population was only permitted to leave their homes under exceptional circumstances, such as for medical care or the procurement of essential food and medication. In stage 3, these restrictions have loosened, and people are allowed to briefly leave their homes for some non-essential activities, however sanitation and hygiene practices, social distancing of 2 meters, and the donning of a mask, must done while in public [11].

With the restrictions introduced by the Risk-Adjusted Strategy, South Africans face work closures, travel restrictions and the banning of large social gatherings, at a scale that has never been experienced before [11]. While these preventative measures are necessary to reduce COVID-19 transmissions, they have inadvertently led to several secondary socioeconomic effects. Work closures have drastically decreased the income of low-skilled workers, while travel restrictions and supply-chain disruptions have led to food insecurity, especially for low-income households [12]. Furthermore, evidence from a global systematic review has revealed that the prevalence of mental health disorders like stress, anxiety and depression have increased by approximately one-third across the general population [13].

Due to these secondary factors and the national and international scale of the restrictions, there is a dearth of evidence to help understand or predict how individuals adhere to the lockdown measures, or if these measures are even effective at reducing the community transmission of COVID-19 in South Africa [14]. Gender differences have been cited as a potential factor affecting adherence, due to pre-existing gender norms and roles. Men may also prioritize the need to provide for their family over adhering to preventative measures [12], while women have shown a higher prevalence of anxiety, depression, and stress during the pandemic [13]. Preliminary reports from the UK have shown that men’s attitudes and behaviors differ from women’s, as they are less likely to wash their hands or wear a mask, and often delay help-seeking [15]. While both genders show different vulnerabilities to the pandemic and associated preventative measures, men have a higher COVID-19 mortality rate of 58%, which reinforces the need to examine gender differences surrounding the pandemic [15].

The International Citizens Project COVID-19 (ICPcovid) is a global consortium of scientists from North and South America, Africa, Asia and Europe, collaborating to investigate the adherence to lockdown restrictions from 22 countries around the world. Aggregate data from all countries will be used to assess the overall effectiveness of adherence to government-recommended measures on the incidence of severe COVID-19 cases and identify the most effective measures to decrease the incidence of severe COVID-19 cases. The specific objective of this study was to describe South Africa’s national adherence to the initially introduced Risk-Adjusted Strategy restrictions, and identify any factors associated with lockdown adherence [16]. This should allow for effective preventative measures to be tailored towards populations that are not sufficiently adhering to the strategy.

## Methods

### Study design

This cross-sectional study was conducted electronically in South Africa from 24 April 2020 until 15 May 2020. Due to the unprecedented national lockdown, a convenience sample with snowballing was used as an online recruitment strategy to achieve the maximum amount of completed surveys. Participants were included if they were residing in South Africa during the lockdown at the time of survey and were able to access the survey using a computer, phone, or other mobile device.

### Survey development

The ICPcovid survey was developed in Belgium by the University of Antwerp [16], and is based on the Citizen Science Corona Survey, which was first launched on 17 March 2020 in Belgium. This survey has been conducted in over 20 countries, with a standard set of questions to define the global response, as well as various questions to evaluate country-specific responses. The survey was adapted for use in South Africa.

### Sampling and data collection

Non-probability sampling was used. Invitations to participate where circulated through an organizational Facebook page and WhatsApp group. A healthcare training and advocacy organization distributed the invitation to all the people registered in their database through a text blast. Lastly, all recipients were asked to forward the invitation to others. Participants anonymously completed the survey via a secured link (https://www.icpcovid.com/en/country/south-africa), which was included in the invitation communication.

**Fig 1:**
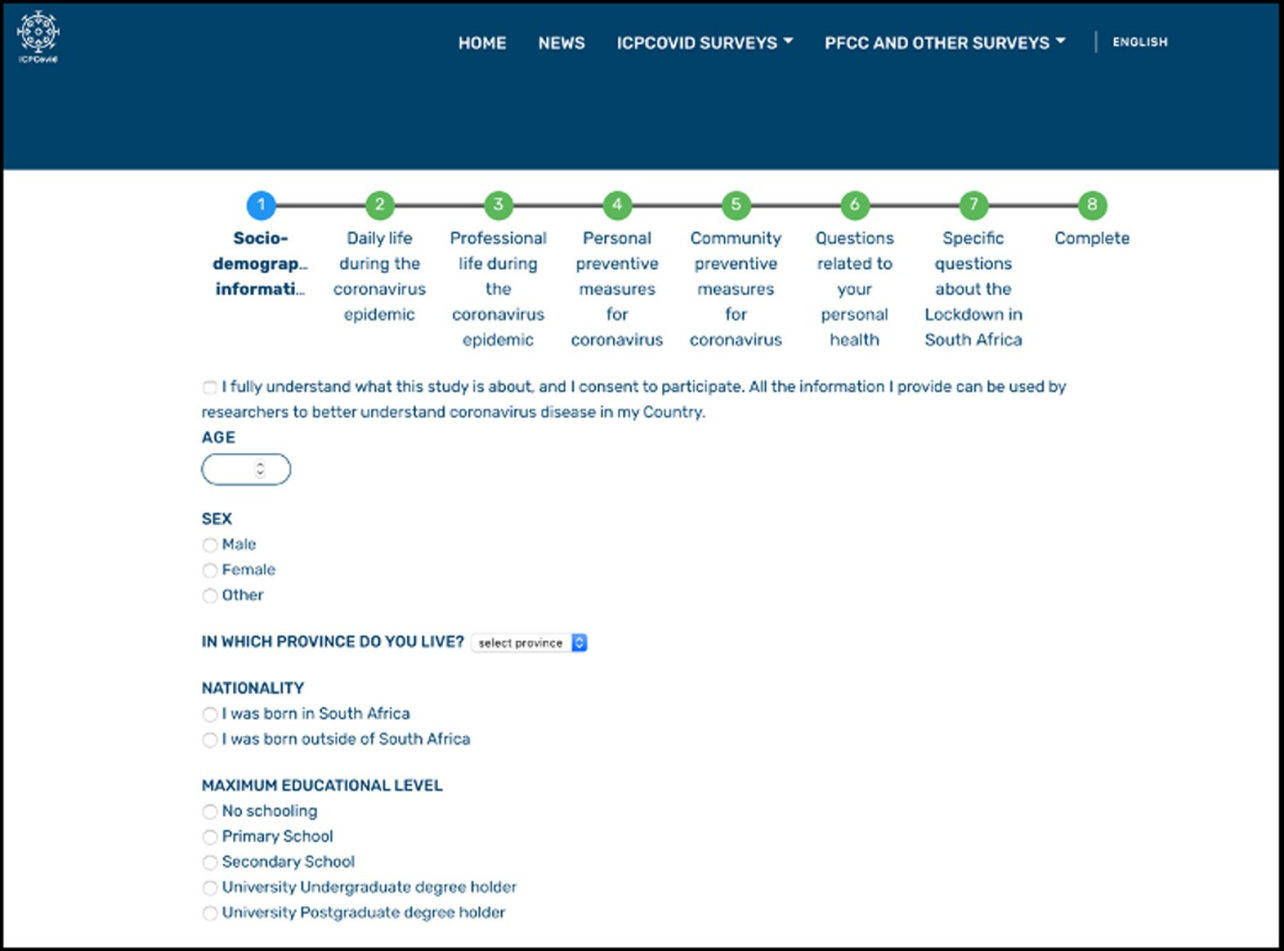
South African ICPcovid-19 survey screenshot.

The South African ICPcovid survey was 46 questions long and consisted of seven sections: sociodemographic; daily life during the epidemic; professional life during the epidemic; personal prevention measures during the epidemic; community preventative measures during the epidemic; personal health questions and South Africa- specific questions (S1 Fig). A screenshot of the survey is presented in Fig 1.

### Data analysis

Data from the survey were cleaned in Excel (Microsoft; Seattle, USA), then exported to Stata V.14 (StataCorp; College Station, USA) for analysis (S1 File). Sociodemographic information and adherence-related questions were described with frequency and percentages. Similar to the analysis of an ICPcovid survey from Brazil, principal component analysis (PCA) of 16 adherence questions was used to calculate a single adherence score for each participant, (with a score of 16 being very high and zero being very low) and a binary variable was created to represent lower lockdown measure adherence versus higher lockdown measures adherence [17].

Unadjusted and adjusted logistic regression analyses were conducted to identify factors associated with lockdown adherence. In the adjusted model, only factors significant in the unadjusted model were included. The statistical significance level was set at p < 0.05 (two-sided).

### Ethical consideration and approval

This survey was conducted as a matter of urgency to examine the South African adherence to COVID-19 lockdown measures and with the lockdown in place, it was not possible to physically recruit for this study or obtain written or oral consent. This methodology was approved by the University of the Witwatersrand Human Research Ethics Committee (non-medical) (reference number 200403), and the introduction to the survey included the following statement: “Participation in the survey is voluntary, you can cancel it at any time without any disadvantages. Your data will be stored anonymously and treated confidentially.”

## Results

### Sample characteristics

The following data is presented in Table 1.

**Table 1:** Demographic characteristics.

Demographic	Frequency(n = 951)	Percentage(%)*
Which province do you live?		
Eastern Cape	13	1.4
Free State	5	0.5
Gauteng	655	68.9
KwaZulu Natal	104	10.9
Limpopo	9	1.0
Mpumalanga	18	1.9
North West	54	5.7
Northern Cape	5	0.5
Western Cape	88	9.3
Nationality		
Born in South Africa	849	89.3
Born outside of South Africa	102	10.7
Age		
0–24 years old	98	10.3
25–54 years old	731	76.9
55–64 years old	87	9.2
65 years or older	35	3.7
Sex		
Female	672	70.7
Male	176	29.0
Other	3	0.3
Maximum educational level		
No Schooling	1	0.1
Primary School	3	0.3
Secondary School	242	25.5
University Postgraduate degree	390	41.0
University undergraduate degree	315	33.1
Marital status		
Cohabitation	60	6.3
Divorced	59	6.2
Legally married	516	54.3
Single	304	32.0
Widow/widower	12	1.3
How many housemates do you have? (yourself not included)		
Adults over 70 years of age	951	40.0
Adults between 18 and 70 years of age	826	34.7
Children between 12 and under 18 years of age	208	8.7
Children under 12 years of age	394	16.6
Do you live in:		
a major city suburb	743	78.1
a rural area/village	27	2.8
a small town	181	19.0
In the last week did you have difficulties in obtaining food?		
No	906	95.3
Yes	45	4.7
In the last week, how worried were you about your health?: 1 = not worried to 5 = extremely worried		
1	352	37.0
2	205	21.6
3	228	24.0
4	82	8.6
5	84	8.8
Have you suffered any form of violence or discrimination because of the measures taken against the coronavirus?		
Discrimination because of my ethnicity, race or nationality	22	2.3
Discrimination because of my ethnicity, race or nationality; No violence or discrimination	1	0.1
Discrimination because of my social/economic status	16	1.7
Discrimination because of my social/economic status; Discrimination because of my ethnicity, race or nationality	2	0.2
Discrimination because of my social/economic status; No violence or discrimination	1	0.1
No violence or discrimination	905	95.2
Physical violence at home	3	0.3
Physical violence outside	1	0.1
What do you do for a living?		
Self-employed	143	15.0
Student	64	6.7
Unemployed	103	10.8
Work for a person, institution or company	532	55.9
Work for the government	109	11.5
Have you received government funding or cash transfers?		
No	912	96.6
Yes	32	3.4
Have you been eating more healthy food such as fruits and vegetables since the coronavirus epidemic started?		
No	422	44.4
Yes	529	55.6
Have you been taking more vitamin tablets since the coronavirus epidemic started?		
No	515	54.1
Yes	436	45.9
Did you have flu-like symptoms in the last 7 days (cough or sore throat, shortness of breath, headaches, body pains, fever, loss of taste or smell)?		
Do not know	38	4.0
No	782	82.2
Yes	131	13.8
Do you smoke?		
No	824	86.6
Yes	127	13.4
Do you have an underlying disease (heart disease, asthma, diabetes, hypertension, cancer, HIV, TB, etc.)?		
Not to my knowledge	733	77.1
Yes	218	22.9
If you did not test or your test results were negative/unknown, are you scared about getting coronavirus?		
No	347	36.5
Yes	604	63.5
Do you feel you are well informed about Covid-19 preventive measures?		
No	32	3.4
Yes	919	96.6
Do you think the lockdown was necessary in South Africa?		
No	44	4.6
Yes	907	95.4
Which emotion did you feel the most during the lockdown?		
Angry	21	2.2
Anxious	292	30.7
Depressed	63	6.6
Happy	182	19.1
Sad	26	2.7
Scared	33	3.5
Tired	110	11.6
Worried	224	23.6

*Percentages may not add up to 100.0% due to rounding.

A total of 951 participants responded to the survey. Participants responded from all provinces, however the more than two-thirds (655; 68.9%) were from Gauteng and 743 (78.1%) lived in a major city suburb. More than three-quarters (731; 76.9%) of the participant were between the ages of 25 and 54, 672 (70.7%) participants were female, 705 (74.1%) had a university degree and 516 (54.3%) were legally married.

In the week prior to survey completion, 45 (4.7%) participants had difficulty obtaining food, 32 (3.4%) had received government funding or cash transfers, and 166 (17.4%) were worried or extremely worried about their health. When asked about nutrition since the epidemic started, 529 (55.6%) and 436 (45.9%) participants stated that they were eating healthier foods and taking more vitamins, respectively. Flu-like symptoms were experienced by 131 (13.8%) participants in the previous week. There were 127 (13.4%) smokers and 218 (22.9%) participants with underlying medical conditions. Nearly all (919; 96.6%) participants felt well informed about COVID-19 preventative measures and 907 (95.4%) felt that the lockdown was necessary in South Africa.

### COVID-19 testing

A total of 82 (8.6%) participants had been tested for COVID-19, and only 1 (1.2%) tested positive. Nearly half (38; 46.3%) of the tests were done at a private laboratory, 20 (24.4%) were done at a public facility, 12 (14.6%) at a hospital, 9 (11.0%) at a doctor’s office, and 3 (3.7%) tested at home (Table 2).

**Table 2:** COVID-19 testing information.

COVID-19 testing questions	Frequency(n = 951)	Percentage(%)*
Were you tested for Covid-19?		
No	869	91.4
Yes	82	8.6
How was the testing done?		
At a private laboratory	38	46.3
At a public facility	20	24.4
At home	3	3.7
At the doctor	9	11.0
In a hospital	12	14.6
What was the test result?		
Do not know	10	12.2
Negative	71	86.6
Positive	1	1.2

*Percentages may not add up to 100.0% due to rounding.

### Adherence measures

For social distancing, 635 (66.8%) participants had no contact with people outside their household, however 147 (15.5%) participants reported physical contact with someone in the last week and 169 (17.8%) participants reported contact with someone longer than one week prior. When leaving the house, 905 (95.2%) followed the 1.5-2m social distancing rule, however 99 (10.4%) participants stated that they had attended meetings or gatherings with more than ten people in the last 14 days and 28 (2.9%) stated that they had been in a vehicle with more than 5 people in the last 14 days. In the previous 14 days, 3 (0.3%) people went to a restaurant, bar or party, 8 (0.8%) participants had attended a religious gathering, 2 (0.2%) participants went to the gym and 12 (1.3%) participants went to a beauty salon (Table 3).

**Table 3:** Adherence measures.

Adherence measures	Frequency(n = 951)	Percentage(%)*
**Social distancing**		
When was the last time you shook hands, gave a kiss or had any form of physical contact with someone other than a housemate?		
Last 3 to 5 days	51	5.4
Last two days	54	5.7
More than one week ago	169	17.8
No contacts with persons outside my household	635	66.8
Today	42	4.4
I follow the social 1.5-2m meters distance rule		
No	46	4.8
Yes	905	95.2
Were you in a meeting or gathering with more than 10 persons during the last 14 days?		
No	852	89.6
Yes	99	10.4
Did you go to a restaurant, bar, club, dancing, party, or concert during the last 14 days?		
No	948	99.67
Yes	3	0.3
Did you go to a religious gathering during the last 14 days?		
No	943	99.2
Yes	8	0.8
Were you in a vehicle or bus with more than 5 persons during the last 14 days?		
No	923	97.1
Yes	28	2.9
Were you in a public gym in the past 14 days?		
No	949	99.8
Yes	2	0.2
Did you go to a beauty parlor, massages, spa, hairdresser or nail studio in the past 14 days?		
No	939	98.7
Yes	12	1.3
**Hygiene measures**		
When I cough or sneeze, I cover my mouth and nose with a tissue paper or into my elbow		
No	32	3.4
Yes	919	96.6
When I cough or sneeze, I usually wash/disinfect my hands immediately afterwards		
No	254	26.7
Yes	697	73.3
I wash my hands using soap and water regularly during the day		
No	44	4.6
Yes	907	95.4
I use a hand sanitizer regularly during the day		
No	269	28.3
Yes	682	71.7
I avoid touching my face (eyes, nose and mouth)		
No	267	28.1
Yes	684	71.9
I wear a face mask when going outside		
No	177	18.6
Yes	774	81.4
**Self-quarantine requirements**		
I stay home when I feel flu-like symptoms		
No	97	10.2
Yes	854	89.8
**Travel Restrictions**		
Did you travel in the past 14 days?		
No Travel	928	97.6
Yes I travelled to other provinces	23	2.4

*Percentages may not add up to 100.0% due to rounding.

In terms of coughing or sneezing, 919 (96.6%) participants covered their mouth and nose with a tissue or their elbow and 698 (73.3%) participants stated that they usually wash/disinfect their hands immediately after. Regular hand washing with soap and water was done by 907 (95.4%) participants while 682 (71.7%) used hand sanitizer regularly. Face masks were worn outside by 774 (81.4%) participants and 684 (71.9%) avoided touching their face throughout the day. Only 23 (2.4%) participants stated that they had travelled out of province the previous 14 days, and 854 (89.8%) stayed in their home when they felt flu-like symptoms.

### Determinants of adherence

Logistic regression analysis identified four predictors for adherence to COVID-19 preventative measures (Table 4). Being male and self-reporting flu-like symptoms were predictors of decreased adherence with an AOR of 0.72 (p = 0.032; CI = 0.528–0.971) and 0.42 (p = 0.000; CI = 0.277–0.628), respectively. Conversely, predictors associated with increased adherence to preventative measures were increased vitamin tablet intake, with an AOR of 1.37 (p = 0.023; 1.044–1.797) and cohabiting or being legally married, with an AOR of 1.39 (p = 0.025; CI = 1.042–1.847).

**Table 4:** Determinants of adherence.

Determinants of adherence	AOR	95% CI	P value
Age			
Less than 25 years old	1	-	-
25–54 years old	0.840	0.522–1.350	0.472
55–65 years old	1.321	0.699–2.496	0.392
Over 65 years old	1.386	0.606–3.167	0.440
Sex			
Female	1	-	-
Male	0.716	0.528-.971	*0*.*032*
Marital status			
Single, divorced or widowed	1	-	-
Cohabiting or legally married	1.388	1.042–1.847	*0*.*025*
Taking more vitamins since epidemic started?			
No	1	-	-
Yes	1.370	1.044–1.797	*0*.*023*
Flu-like symptoms in the last 7 days?			
No flu-like symptoms	1	-	-
Experienced flu-like symptoms	0.417	0.277-.628	*0*.*000*
Do you smoke?			
No	1	-	-
Yes	0.712	0.475–1.066	0.099
Do you have an underlying disease?			
No	1	-	-
Yes	0.822	0.587–1.151	0.253
Fear of getting COVID-19?			
No	1	-	-
Yes	1.084	0.802–1.465	0.601
Felt well informed?			
No	1	-	-
Yes	1.027	0.458–2.303	0.948
Perceived lockdown as necessary?			
No	1	-	-
Yes	1.477	0.760–2.874	0.250
Worried about health during lockdown?			
No	1	-	-
Yes	1.096	0.813–1.476	0.548

AOR-adjusted odds ratio; CI-confidence interval

## Discussion

Predictive modeling has been done to forecast the spread of COVID-19 in South Africa [18, 19], however this study is believed to be the first to evaluate pragmatic preventative measures. Participants reported generally high adherence to preventative measures, especially social distancing of 1.5-2m and regular handwashing, however mask use was lower. The stronger adherence to preventative measures by females is in-line with results from the UK, Brazil and China [15, 17, 20], however the increased adherence by participants who have upped their vitamin intake is unique to South Africa. The decreased adherence by participants experiencing flu-like symptoms was also unique to South Africa and is concerning for the spread of COVID-19. Marital status was not a predictor in Brazil; however, it was in China, only when legally married was compared to all other statuses [17–20].

Only 81.4% of participants donned a mask while going outside, which was much higher than the 45.5% reported in the Brazilian ICPcovid survey [17], however it was much lower than in China where 98.0% of respondents to a similar online survey reported using a mask [20]. Misconceptions surrounding the use of face masks have been reported early on in the outbreak, as many people thought they were for personal protection, not for preventing the possible spread from asymptomatic people [21]. In South Africa, the *Disaster Management Act*: *Regulations*: *Alert level 3 during Coronavirus COVID-19 lockdown*, downloadable from www.sacoronavirus.co.za mandates face mask (preferably cloth) usage in public, however face masks are not mentioned on the website’s homepage, which has prevention tips, that include frequent handwashing, avoiding the touching one’s face and distancing from sick people [11]. In Africa, face masks have been viewed as a modern medical comfort, and in some regions, prices have surged 600% due to increased demand. This scarcity and increase in price reinforce face masks as a luxury item, so along with this increased awareness, masks must be also normalized and made available to the entire population [22].

The decreased adherence by participants experiencing flu-like symptoms, single people and males may be contextualized by South Africa’s fragile economy, as one month after the lockdown measures were implemented, a 40% reduction in active employment was observed [23]. This drastic employment loss has shocked household incomes and shifted priorities towards essentials such as food security [12]. For those still employed, the burden to provide for one’s family may outweigh the desire to stay home due to flu-like symptoms [12]. Similarly, single people may be less likely to adhere to preventative measures, as there is no one else to provide for them, and the burden falls solely on themselves [12].

In South Africa, the national workforce is 56% male, and females dominate low-education occupations, such as domestic work, clerk, technician, and the informal sectors [24]. The National COVID-19-related employment loss disproportionately affected females, especially manual and informal workers, people with minimal education and poor people [23], and with more females out of work, the males that are still employed may feel a greater burden to prioritize the need to provide for their family over adhering to preventative measures [12]. Attitude and behavior research also suggest that males are less likely to engage in public health measures than females, despite their increased risk of mortality [15].

Sex-disaggregated data thus far has shown no difference in COVID-19 prevalence. However, disproportionately, more males are dying from the disease, possibly due to higher prevalences of smoking and comorbidities and sex-based antiviral immunity expression [25–27]. Alternatively, front-line healthcare workers are predominantly female, as are often the caregivers in the family, which has led to higher disease prevalence in previous pandemics, such as the 2014 Ebola outbreak in West Africa [27].

The nation-wide layoffs, in conjunction with the lockdown measures, have also spawned a mass of internal migration where an estimated 5–6 million people have moved back to their home villages from urban townships between March and May 2020 [28]. While the cost of living may be lower in hometowns, this is not sustainable, as most employment opportunities are in urban areas, which will lead to more travel once savings run out, especially during a prolonged lockdown. An Italian study found that compliant respondents were less willing to increase their self-isolation efforts when presented with a long hypothetical extension to lockdown measures [29]. With no vaccine availability for total population rollout, the South African government must continue to communicate the importance of preventative measures to avoid lockdown fatigue, as predictive modelling suggests that a relaxation of social distancing measures by just 2% may lead to a 23% rise in cumulative cases [19].

### Limitations

Due to the lockdown conditions, this survey was conducted online with a convenience sampling, which may present a selection bias, from the mobile and data requirements needed to access the survey. Furthermore, three-quarters of respondents were university graduates, which does not reflect the general population of South Africa. These findings also only apply to people with internet access, so further studies must be done to include those without internet access, especially in remote rural areas.

While the global ICPcovid study has four objectives, this study only investigated national adherence to preventative measures and the associated predictors of adherence. There was only one participant that tested positive for COVID-19, so the overall effectiveness of adherence on the incidence of COVID-19 cases, and the identification of effective measures to decrease said incidence, could not be evaluated.

Lastly, although posing important questions, this survey design was not guided by a theoretical framework. Further research investigating COVID-19 prevention, management and risk behaviors should be underpinned by relevant theoretical and conceptual models to generate pertinent information for policy development and maximum social impact.

### Public and social policy implications

As many are currently living from month to month (even day to day for those not in full time employ), people will continue to pursue employment, regardless of flu-like symptoms, and especially for single people and males, who bear the brunt of the burden to provide particularly during financially constrained circumstances [12]. Additional to ensuring available and usable hygiene and sanitization services, countries need to implement well controlled social support programs and have readily available personal protective equipment for all, especially individuals with the burden to provide for a family, like males, and those who are more likely to die from COVID-19 [25–27]. A review of national health policies by the UNAIDS found that the health of adolescent and adult males were not adequately addressed in these policies [27, 30], and to properly do so, especially regarding COVID-19, accurate sex-disaggregated data must be readily reported [27].

## Conclusion

On day 29 of lockdown (April 24, 2020), when this survey was first distributed, there were 3954 cases and 75 deaths, however by February 7, 2021, South Africa has nearly 1.5 million infections and over 46000 deaths [3]. While this study has identified sub-optimal mask usage and decreased adherence to preventative measure by males, single people and symptomatic individuals, as priority areas for a strengthened health response, these factors alone do not solely account for the steadily increasing incidence of COVID-19 cases. The government must continue to communicate the importance of effective preventative measures, and more general population studies with sex-disaggregated data must be conducted to identify other adherence predictors and areas of improvement, across all socioeconomic demographics of the country.

## Supporting information

S1 Fig ICP COVID-19 survey. (XLSX)

S1 File ICP Covid-19 dataset. (DOCX)
