# Supplementary material for: International citizen project to assess early stage adherence to public health measures for COVID-19 in South Africa
Source: PLoS One. 2021 Mar 4;16(3):e0248055. doi: 10.1371/journal.pone.0248055 (PMC7932542; doi:10.1371/journal.pone.0248055)
Supplement: S1 File — (DOCX) [file pone.0248055.s002.docx]

**S1 File. ICP COVID-19 survey**

**How do you deal with Corona?**

**Introduction**
The government took several measures recently to prevent the spread of coronavirus (Covid-19). With this short questionnaire, we are trying to find out how you experience and apply the instructions implemented against coronavirus. We call upon volunteers to complete this questionnaire as truthfully as possible; this helps us to investigate which measures are effective in these exceptional times. It should take you less than 10 minutes to answer the questions.

Participation in the survey is voluntary, you can cancel it at any time without any disadvantages. Your data will be stored anonymously and treated confidentially.

This is an online, voluntary survey initiated by researchers based at Ezintsha, a sub-division of the Wits Reproductive Health and HIV Institute, a division of the Wits Health Consortium, in South Africa. This survey is being conducted in partnership with the Global Health Institute at the University of Antwerp and is being conducted in over 20 countries. If you receive this survey and are not currently living in South Africa, we ask that you **do not** complete it. We do not require your personal information, and the data will be used to understand the feasibility and effectiveness of implementing preventive measures for the coronavirus at the individual level. If you would like to know more about our privacy policy, please visit [www.icpcovid.com](http://www.icpcovid.com.) or contact [coronasurveysa2020@gmail.com](mailto:coronasurveysa2020@gmail.com).

Q0 Date: _______________

Q1 Age?

- <20
- 20-30
- 31-40
- 41-50
- 51-60
- 61-70
- 70-80
- >80

Q2 **What's your gender?**

- Male
- Female
- Other

Q3 **In which province do you live?** ________________________

Q3b: **Do you live**

- in a rural area/village,
- a suburb
- a town?

Q3c: **Do you live in**

- a house,
- a house with a garden,
- an apartment,
- an informal settlement?

Q4 **How many people live in the same house as you (not counting yourself)? Please write the number of persons for each age group.**

- Adults over 70 years of age: ________________
- Adults between 18 and 70 years of age: ________________
- Children between 12 and under 18 years of age: ________________
- Children under 12 years of age: ________________

Q5 **How many people apart from those living with you did you talk to yesterday face to face (not by phone, chat etc.)?**

Q6 **Was yesterday a weekday or a weekend day?**

- Weekday
- Weekend

Q7 **When was the last time you shook hands, gave a kiss, or had any form of physical contact with someone other than a person living with you?**

- Yesterday
- 2 days ago
- More than 2 days ago
- I have not had physical contact with anyone outside of my house

Q8 **What's your current occupation?**

- None
- Student
- Worker from home
- Worker in an open space (market, shop, roadside, etc.)
- Worker in a closed office
- Work in other people’s home

Q9 **Are you in the healthcare industry (as a student or worker)?**

- Yes
- No

Q10 **How many days per week do you usually go to school or work? ________________**

Q11 **How many days did you go to school or work last week? ____________________**

Q12 **Are you working from home today?**

- Yes
- No
- Not applicable

*If No to question 12*

Q13 **Why are you not working from home this week?**

- Not possible with my job
- It is possible, but is not allowed by my employer
- I'm at home but not working
- Other ________________________________________________

*Display the following Question 14  If for Question 4 the answer is Children between 12 and 18 or children >12*

Q14 **How did you arrange the care of your children today?**

- At home on their own
- At home with other housemates
- To school/childcare
- At friends / acquaintances
- At aunts or uncles
- At grandparents
- other

Q15 **Did you have flu-like symptoms in the last 7 days (cough or sore throat, shortness of breath headaches, body pains, fever)?**

- Yes
- No
- Do not know

*If yes go to question 16 and 17 if no go to question 20*

Q16  **How many days have you had flu-like symptoms?** _______________

Q17 **Are these symptoms still present?**

- Yes
- No

*If yes go to question 18 If no go to question 19*

Q18 **Have any of your housemates had flu-like symptoms in the last 7 days?**

- Yes
- No
- Do not know

Q19 **How many days ago did the symptoms disappear?** ___________________

Q20 **On a scale of 0 to 10, can you indicate the extent to which people in your environment have practically changed their behaviour to the government recommendations?**

*Zero=no changes, 10=very strong change***: _________**

*Q21 This section will be about the general restrictive behaviors*

21.1 **Approximately how many times have you been washing your hands or using sanitizer a day?**

- None
- 1 time
- 2 times
- 3 or more times

21.2 **Have you been in a meeting or gathering with more than 100 persons during the last 14 days?**

- Yes
- No

21.3 **Have you been to a meeting or gathering of more than 10 persons during the last 14**

- Yes
- No

21.3 **Have you been in a restaurant, bar, club, dancing during the last 14 days?**

- Yes
- No

21.4 **Have you been in a vehicle with more than 5 persons during the last 14 days?**

- Yes
- No

21.5 **Have you been in a public gym in the last 14 days**?

- Yes
- No

21.6 **Have you been to the beauty parlour massages, spa, hairdresser or nail studio in the past 14 days**?

- Yes
- No

21.7 **Have you been in the fresh market, live animals, or contacted animals in the past 14 days**?

- Yes
- No

21.8 **Have you been** **using individual spoons and plates when eating together with family members in the past 7 days** ?

- Yes
- No

21.9 **Have you been** **using individual spoons and plates when eating together with family and non-family members in the past 7 days** ?

- Yes
- No

21.10 **Did you** **travel to other provinces or abroad in the past 14 days**?

- Yes
- No

*This section is about personal protective measures*

Q22 **Which protective measure are you using**?

I follow the social 2 meters distance rule

- Yes/ No

I wear a face mask when going outside

- Yes/ No

I cover mouth and nose when coughing or sneezing with paper tissue and wash my hands afterwards

- Yes/ No

I measure my body temperature

- Yes/ No

I regularly wash my hands

- Yes/ No

I use a hand sanitizer

- Yes/ No

I avoid touching my face (eyes, nose and mouth)

- Yes/ No

I disinfect my phone

- Yes/ No

I eat healthy food such as fruits and vegetables more often

- Yes/ No

I take supplement vitamins and minerals

- Yes/ No

Q25 **Do you have an underlying disease (e.g. heart disease, diabetes, hypertension, cancer, HIV, tuberculosis, etc)?**

- Yes
- No
- Do not know

*If you have an underlying disease go to question 26 if not got to question 27*

Q26 **If you have an underlying disease did you experience difficulties to obtain your medication?**

- Yes
- No

Q27 **Do you smoke?**

- Yes
- No

*Go to question 28*

Q28 **During the last week did you have difficulties in obtaining food?**

- Yes
- No

*If yes go to question 29 if no go to question 30*

Q29 **What was the reason you had difficulties in obtaining food last week?**

- No money
- Little food available in shops, market
- I felt it was unsafe to go out to buy food
- I was too ill to go out

*Go to question 30 Stress related questions*

Q30 **During the last two weeks, how often did you feel worried or afraid about your health?**

- Not at all
- Several days
- More than half the days
- Nearly every day

Q31 **How difficult is it for you personally to follow a protective measure of staying as much as possible in your home? On a scale of 1 (not difficult at all) to five (extremely difficult)**

- 1
- 2
- 3
- 4
- 5

*This section is specifically about the Lockdown in South Africa*

**Q32 How often do you normally use public transport?**

- Once a day on weekdays
- Twice a day on weekdays
- On weekends only
- A few times a week
- I have my own vehicle

**Q33 How often did you use public transport during the lockdown in South Africa?**

- Once a day
- Twice a day
- A few times a week
- I did not use public transport during the lockdown

**Q34 How many times did you leave your house during the lockdown in South Africa?**

- Once a day
- Multiple times a day
- A few times a week
- I did not leave the house

**Q35 Why have you been leaving the house**

- Shopping for essentials
- Exercise
- Work
- My living space is small and I wanted to get away
- Not applicable

**Q36 Which best describes your grocery shopping just before the lockdown?**

- Stocked up on everything
- Stocked up on essentials
- No stockpiling – just normal shopping
- No stockpiling because I can go and get food during the lockdown whenever I need it
- Not buying too much – doing online shopping during the lockdown

**Q37 Were you tested for COVID-19**

- Yes
- No

**Q38 How was the testing done?**

- At home
- At the doctors
- At a private laboratory
- At a public facility
- In hospital
- Not tested

**Q39 If yes, was the test positive?**

- Yes
- No
- Don’t know

**Q40 If you did not test or did not have a positive result are you scared about getting coronavirus?**

- Yes
- No

**Q41 If you were to develop fever or a cough, would you know who to contact for medical advice**

- Yes
- No

**If yes, who would you contact?** _______________

**Q42 Do you feel you are well informed about COVID-19 preventive measures?**

- Yes
- No

**Q43 Select which platforms you are getting most of your COVID-19 related information from. Choose your top 3 sources:**

- WhatsApp
- TV
- Radio
- Newspaper
- Friends
- Family
- Social Media such Twitter and Facebook
- Internet searches and websites

**Q44 Do you think the lockdown was necessary in South Africa?**

- Yes
- No

**Q45 During the lockdown did you ever feel (select as many as you like):**

- Happy
- Sad
- Angry
- Anxious
- Depressed
- Scared
- Worried
- Tired

**Q46 Which one in Q45 did you feel the most during the lockdown?**

- Happy
- Sad
- Angry
- Anxious
- Depressed
- Scared
- Worried
- Tired

Thank you very much for your participation.

We would like to invite you to participate in another survey in one week. Participation is voluntary and optional.

Further information about Covid-19 please visit website [www.sacoronavirus.co.za](http://www.sacoronavirus.co.za)

The survey team from Ezintsha and the Global Health Institute.
